# Evaluation of Heat-Treated AISI 316 Stainless Steel in Solar Furnaces to Be Used as Possible Implant Material

**DOI:** 10.3390/ma13030581

**Published:** 2020-01-26

**Authors:** Ioan Milosan, Monica Florescu, Daniel Cristea, Ionelia Voiculescu, Mihai Alin Pop, Inmaculada Cañadas, José Rodriguez, Cristina Aurica Bogatu, Tibor Bedo

**Affiliations:** 1 Faculty of Materials Science and Engineering, Transilvania University of Brasov, 1 Universitatii Street, 500068 Brasov, Romania; milosan@unitbv.ro (I.M.); daniel.cristea@unitbv.ro (D.C.); mihai.pop@unitbv.ro (M.A.P.); bedo.tibor@unitbv.ro (T.B.); 2 Faculty of Medicine, Transilvania University of Brasov, 1 Universitatii Street, 500068 Brasov, Romania; 3 Faculty of Engineering and Management of Technological Systems, Politehnica University of Bucharest, 313 Splaiul Independentei Bld., 060042 Bucharest, Romania; ioneliav@yahoo.co.uk; 4 CIEMAT-Plataforma Solar de Almeria; Ctra. Km, 4, P.O. Box 44, 04200 Tabernas, Spain; i.canadas@psa.es (I.C.); jrodriguez@psa.es (J.R.); 5 Faculty of Product Design and Environment, Transilvania University of Brasov, 1 Universitatii Str., 500068 Brasov, Romania; cristina.bogatu@unitbv.ro

**Keywords:** 316 stainless steel, solar energy, hyper-hardening treatment, tempering, corrosion resistance

## Abstract

The appropriate selection of implant materials is very important for the long-term success of the implants. A modified composition of AISI 316 stainless steel was treated using solar energy in a vertical axis solar furnace and it was subjected to a hyper-hardening treatment at a 1050 °C austenitizing temperature with a rapid cooling in cold water followed by three variants of tempering (150, 250, and 350 °C). After the heat treatment, the samples were analyzed in terms of hardness, microstructure (performed by scanning electron microscopy), and corrosion resistance. The electrochemical measurements were performed by potentiodynamic and electrochemical impedance spectroscopy in liquids that simulate biological fluids (NaCl 0.9% and Ringer’s solution). Different corrosion behaviors according to the heat treatment type have been observed and a passivation layer has formed on some of the heat-treated samples. The samples, heat-treated by immersion quenching, exhibit a significantly improved pitting corrosion resistance. The subsequent heat treatments, like tempering at 350 °C after quenching, also promote low corrosion rates. The heat treatments performed using solar energy applied on stainless steel can lead to good corrosion behavior and can be recommended as unconventional thermal processing of biocompatible materials.

## 1. Introduction

Biomaterials are synthetic materials that can be used to replace parts of a living system or to ensure the functionality of organs in close contact with living tissue, being able to replace functions lost due to a disease, as a support in the process of healing and for the improvement or correction of the functions of some organs.

According to L.T. Kuhn [1], the most widely used class of biomaterials is that of metallic biomaterials, and stainless steels are among the material categories of this class.

The microstructure of the Cr-Ni-Mo (316 AISI) standardized stainless steel, with high content of Cr (20.50 wt%) and Ni (12.40 wt%) and added Mo (2 wt%) and Cu (0.26 wt%), consists predominantly of highly alloyed austenite, stable even at very low temperatures without transforming into other nonequilibrium phases. This microstructural stability has enabled the use of this alloy for a long time in medical applications, for implant elements (screws, nuts, or rods), for the purpose of temporary fixation until the healing of fractures or for the replacement of joints [2,3].

To improve the corrosion behavior of austenitic stainless steels, a number of technological procedures can be applied: electropolishing [3], heat treatment [4,5,6], surface treatment, or patterning [6,7]. Heat treatments are widely used to obtain mechanical or tribological characteristics adapted to the specific requirements of medical devices [8].

Concentrated solar energy used for heat treatment of metals exhibits increased interest in recent years, but only recently has been dedicated to industrial applications [9,10,11,12]. Certain treatments performed using solar energy allow the reduction or removal of corrosion resistance reduction compounds, such as sigma (σ) and delta-ferrite (δ) [5,13,14], also contributing to the reduction of friction [15]. To emphasize the special effects of the solar energy treatments, heat treatments were performed in electric furnaces using the same values of the process parameters. The experiments carried out using samples from the same alloy showed that heat treatments using solar energy have led to reduced friction coefficient values, allowed the dissolution of the embrittling compounds and resulted in a decrease of the corrosion rate, all compared to the samples produced by conventional means. This behavior may be due to the much higher heating rates obtained using the solar installation, the absence of direct interaction between the alloy and the furnace hearth, and the reduced dimensions of the treated samples [14]. The disadvantages of the solar energy treatment method are related to higher costs (expensive installations), geographical positioning (they should be located only in areas where solar irradiation is intense), and weather conditions [15].

A common method used to improve the mechanical, chemical, and functional characteristics of biocompatible highly alloyed steels is to modify the standardized chemical composition. The addition of Mo confers better stability at high temperatures [16], while the simultaneous addition of Co, Cr and Mo leads to increased corrosion resistance at ambient or high temperatures [17,18,19,20].

The resistance against pitting corrosion of stainless steels is insufficient in aggressive media containing S^2−^, Cl^−^, or other halide anions. Although a natural passive film forms in situ on the alloy surface, it cannot effectively protect the material, thus the need for further corrosion resistance improvement procedures, especially in the case of implant materials [20,21].

The corrosion behavior in various media is of great importance [21,22,23], as the release of chemical elements from the implant into the biological medium can lead to problems in the implantation tissues (necrosis, local concentrations exceeding acceptable limits, the occurrence of tumors, allergic reactions, etc. [1]).

In this paper, the results on the influence of the concentrated solar energy used for heat treatments on the characteristics of 316 AISI-modified stainless steels are presented. The electrochemical characterization of the samples was used to determine their corrosion resistance in NaCl 0.9% and Ringer’s solution, which imitate biological fluids.

Microstructural changes related to the phase transformations obtained after heat treatments, as well as changes in mechanical characteristics (microhardness), consistent with the electrochemical test results, highlight the beneficial effects of solar energy heat treatments, coupled with chemical composition alternations.

## 2. Materials and Methods

The starting material used for the study was 316 AISI stainless steel, whose chemical composition was determined using Spectromax XF-B. Its chemical composition was modified as compared to the standardized commercial grade by the addition of 0.26 wt% Cu, to enhance the corrosion resistance and to increase the austenitizing effect of nickel (Table 1) [17,18].

Five cylindrical samples (Φ10 × 5 mm) were cut from the modified alloy ingots. The samples were coded as follows; Sample A—untreated 316 steel, and Samples B, C, D, E–316 are alloy heat-treated using concentrated solar energy, using various process values (the sample codes and the processing parameters are presented in Table 2).

**Figure 1:**
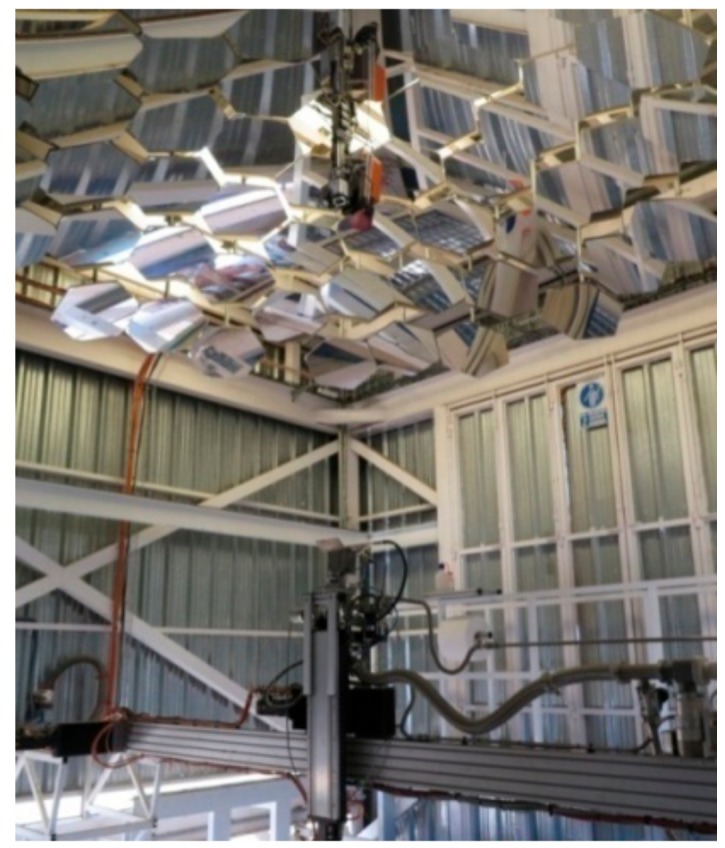
SF5 vertical axis solar furnace (test room).

The samples were heated using solar energy in the vertical axis solar furnace SF5 of CIEMAT, Plataforma Solar de Almeria in Tabernas (Spain). The 5-kW vertical axis solar furnace can provide a maximum flux density peak of 7000 kW/m^2^ and focus spot of 25 mm in diameter [24,25,26,27,28]. The main components of the vertical axis solar furnace (Figure 1) (VSF) are: heliostat, concentrator, attenuator, and test table.

**Figure 2:**
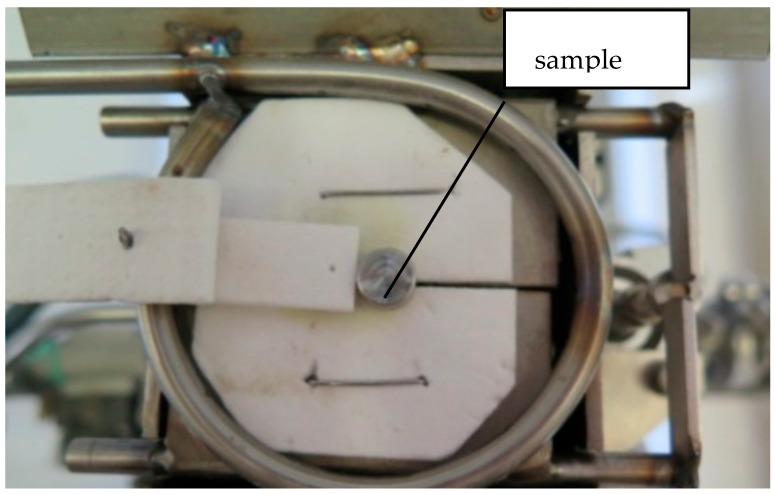
Sample placed on the heating table. The latches open at the desired temperature, and the sample falls into the quenching medium.

The samples were placed on the test table, under Ar protective atmosphere (Figure 2). A thermocouple (TC) was placed into a machined hole of the metallic sample and a solar-blind IR camera was used for temperature control during the heating process.

The samples were subjected to an “immersion quenching” heat treatment starting with heating at 1050°C, followed by rapid cooling in water (approx. 24 °C). Moreover, the samples were tempered at different temperatures, namely, 150, 250, and 350 °C (Table 2). Note that the heating rate for all the samples (B to E) to reach the austenitizing temperature was 210 °C/min, and the time allocated for heating the samples (B–E) up to the tempering temperature was of about 3 minutes.

**Figure 3:**
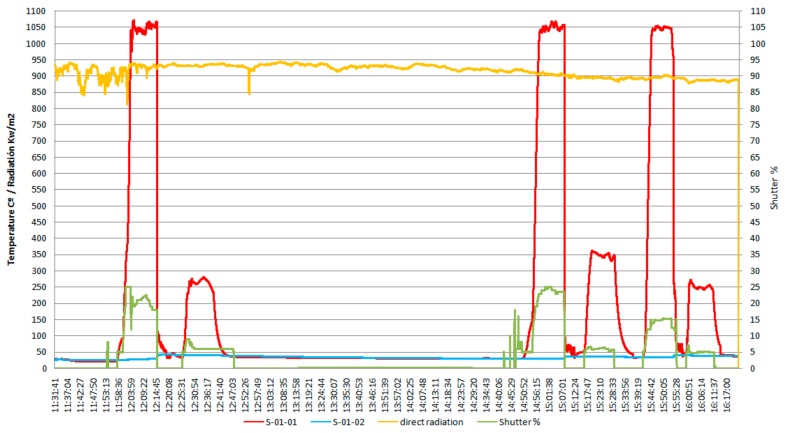
The specific diagram for the heat treatments applied to the AISI 316 modified stainless steel samples.

According to the data shown in Figure 3, which shows the variation of sample temperature, the direct irradiation, and shutter opening percentage, all as function of time, the average heating rate to reach the austenitization temperature was of ~3.5 °C/s, while the average heating rate for tempering was of ~1.5 °C/s, and the value of the solar radiation (Direct Normal Irradiance (DNI)) varied between 850 and 950 kW/m^2^.

In the heating diagram (Figure 3) the meaning of the codes used is as follows:

- S-01-01 (red curve): evolution of the specific temperature during the heat treatment (quenching followed by tempering).

- S-01-02 (blue curve): temperature variation of the cooling water.

- Variation of the direct radiation (incidental solar radiation), in kW/m^2^ (yellow curve).

- The opening angle percent during the sample irradiation (Shutter –SPI), in % (green curve).

The tests related to chemical composition, structural evaluation and microhardness tests were conducted at Transilvania University of Brasov (Brasov, Romania). Several measurements were performed on each sample, the results were averaged, and the standard deviation was calculated.

The X-ray diffraction patterns were recorded on a Bruker AXS D8 discover X-ray Diffraction (XRD) using a monochromatic Al_Kα_ X-ray source, to identify the phases and constituents occurring as function of the heat treatment conditions. The sample microstructure was assessed by scanning electron microscopy (SEM) at the Laboratory for Metallography Testing (LAMET) of the POLITEHNICA University of Bucharest (Bucharest, Romania). 

The electrochemical corrosion tests were conducted at the Laboratory of Biophysics, the Faculty of Medicine, from Transilvania University of Brasov. The measurements were performed for all stainless-steel samples in a conventional electrochemical cell, containing three electrodes: discs of modified AISI 316 stainless steels, as working electrode with an active surface area of 0.785 cm^2^; platinum wire as auxiliary electrode; Ag/AgCl/3.5 M KCl as reference electrode. The working electrode was isolated by embedding it in epoxy-acrylic resin. Before electrochemical measurements, the samples were ultrasonically cleaned in distilled water and rinsed with ethanol. The potentiodynamic methods and electrochemical impedance spectroscopy (EIS) were carried out both in 0.9% NaCl and Ringer’s solutions at room temperature (22 °C) by using a PC-controlled potentiostat PalmSens3 (Palm Instruments BV, Houten, The Netherlands) with PSTrace 5.5 software (Palm Instruments BV, Houten, The Netherlands). The electrochemical measurements were repeated three times in each case and then the average values of all corrosion parameters were calculated considering the computed standard deviation values. The potentiodynamic tests were recorded at a 0.002 V/s scan rate. 

The tests were interrupted at certain values of current density, indicated by the abrupt increase plots, where the samples reached the stable pitting regime. During EIS measurements, an RMS perturbation of 10 mV was applied over the frequency range 50 kHz to 0.1 Hz, with 10 frequency values per frequency decade. Using the PSTrace 5.5 software with FRA module, the electrical parameters values of the electric equivalent circuit that best match the experimental data were inferred.

## 3. Results 

### 3.1. Microstructure

To reveal the microstructure aspects, the samples were mirror polished (using abrasive grit paper and powders) and then were subjected to etching using aqua regia for 10 seconds.

**Figure 4:**
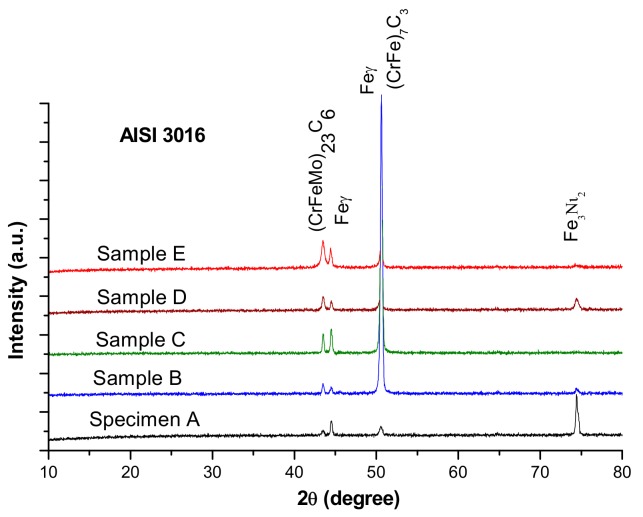
X-ray diffraction patterns of AISI 316 modified stainless steel samples.

According to the Schaeffler diagram, the microstructure corresponding to the chemical composition of the experimental steel contains austenitic matrix (Feγ), typical for untreated highly alloyed chromium steels, and gated islands of delta ferrite (Feδ) surrounded by very thin carbide networks. The X-ray diffraction patterns of AISI 316 stainless steel samples, presented in Figure 4, show complex chromium and molybdenum carbides (M_23_C_6_ (CrFeMo)_23_C_6_ and M_7_C_3_ (CrFe)_7_C_3_). 

**Figure 5:**
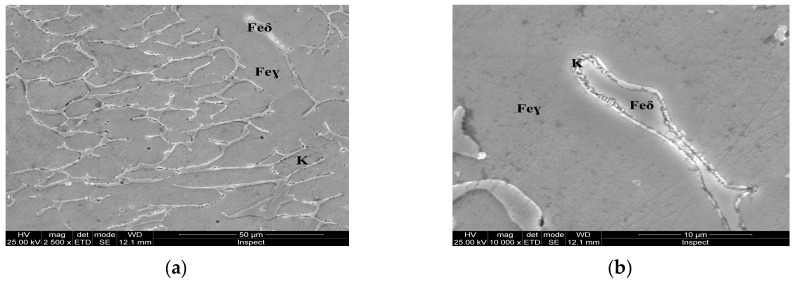
Microstructural aspects of the AISI 316 modified stainless steel sample in the as-cast state (Sample A): (**a**) ×2500; (**b**) detail of the Feδ phase, ×10,000.

**Figure 6:**
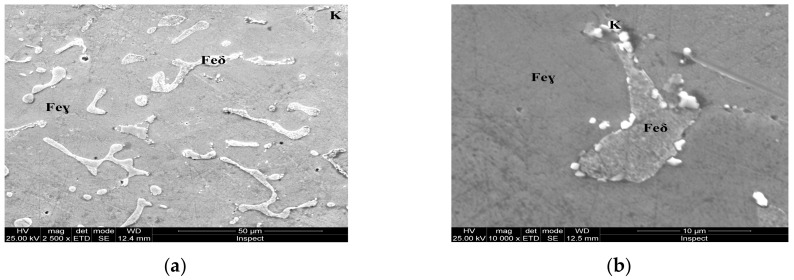
Microstructure of the AISI 316 modified stainless steel sample, heat-treated (Sample B): (**a**) Feγ base matrix, Feδ phase, and carbides (K), ×2500; (**b**) detail of the Feδ phase surrounded by carbides, ×10,000.

**Figure 7:**
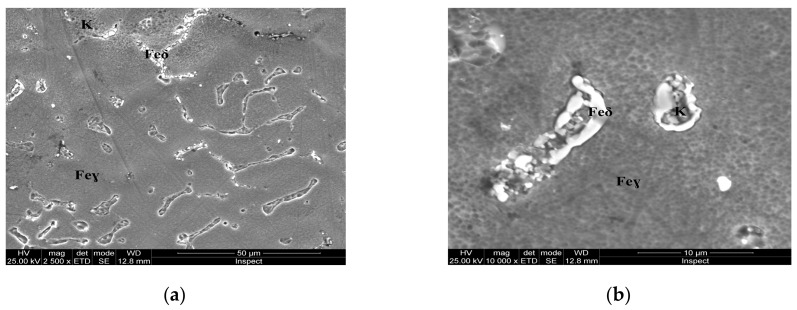
Microstructure of the AISI 316 modified stainless steel sample, heat-treated (Sample C): (**a**) Feγ base matrix, Feδ phase and carbides (K) ×2500; (**b**) detail of the Feδ phase surrounded by larger carbides, ×10,000.

**Figure 8:**
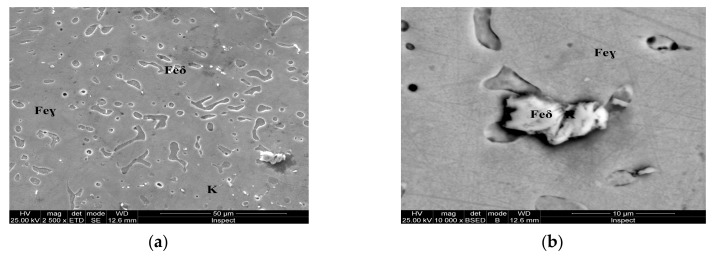
Microstructure of the AISI 316 modified stainless steel sample, heat-treated (Sample D): (**a**) Feγ base matrix, Feδ phase and small carbides (K) ×2500; (**b**) detail of the Feδ phase surrounded by larger carbides, ×10,000.

**Figure 9:**
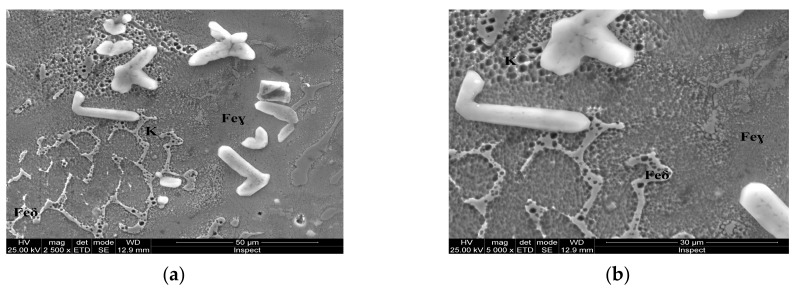
Microstructure of the AISI 316 modified stainless steel sample, heat-treated (Sample E): (**a**) Feγ base matrix, Feδ phase, and coarse carbides (K) ×2500; (**b**) detail of the coarse carbides, ×10,000.

For the as-cast sample, the carbides (K) morphology is represented in the SEM microscopy images (Figure 5). Carbides with eutectic appearance are displayed also at the boundaries of the austenite grains (Feγ) (Figure 5a) or surrounding the Feδ phases (Figure 5b). The microstructural aspects of heat-treated samples B–E are presented in Figure 6, Figure 7, Figure 8 and Figure 9. In all these microstructures, one can observe the austenitic matrix (Feγ) with chromium-rich and molybdenum-rich fine complex carbides (K), M_23_C_6_ (CrFeMo)_23_C_6_ type and M_7_C_3_ (CrFe)_7_C_3_ type, and also small quantities of ferrite (Feδ), as islands.

### 3.2. Hardness Tests

**Figure 10:**
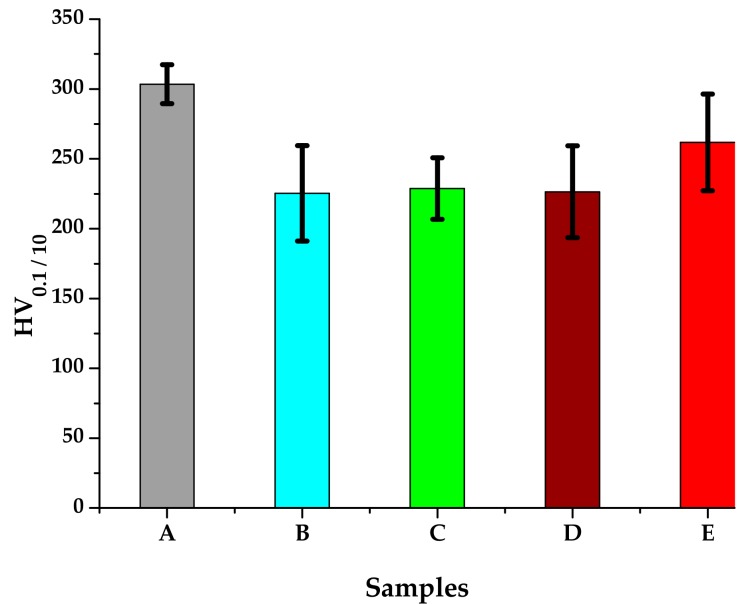
HV_0.1/10_ average hardness of the 316 AISI modified stainless steel samples.

The microhardness measurements were performed using a FM-700 AHOTEC tester (Future-Tech Corp, Talkpier Kawasaki, Kanagawa, Japan). Vickers Hardness (HV 0.1/10) values for the 5 experimental samples are shown in Table 3 and the hardness evolution is plotted in Figure 10. The standard deviation reaches relatively large values, indicating the inhomogeneity of the metallic material after the heat treatments.

The analysis of the hardness test results presented in Table 3 and Figure 10 shows that all heat-treated samples, regardless of the process parameter values, reach lower hardness values than those of the untreated (as-cast) A sample. 

### 3.3. Electrochemical Evaluation

**Figure 11:**
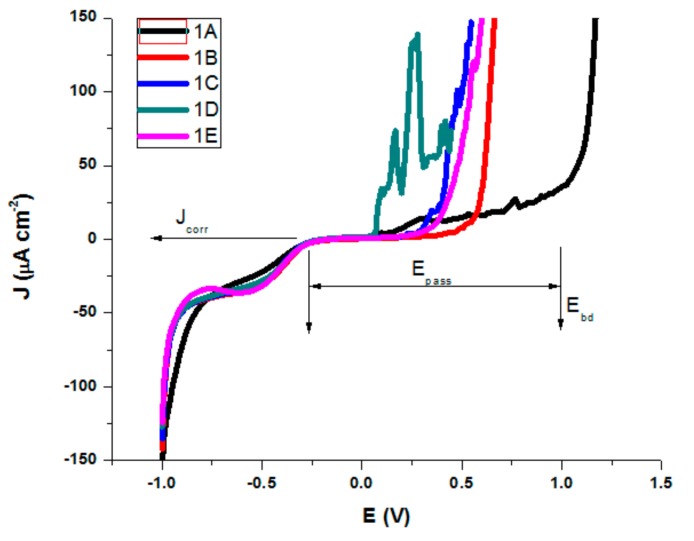
Polarization curves in NaCl 0.9% for AISI 316 modified stainless steels.

The electrochemical characterization of the samples was done through potentiodynamic studies and electrochemical impedance spectroscopy (in NaCl 0.9% and Ringer’s solution). Ringer’s solution used for electrochemical characterization contains besides NaCl 0.9%, several other salts dissolved in water, like KCl (0.03%) and CaCl_2_ (0.05%). A tendency of initial experimental growth of current density was observed initially, as can be seen in Figure 11. 

Similar behavior was observed for all treated AISI 316 modified stainless steel samples in Ringer’s solution, but with a slight difference in corrosion parameters. The testing parameters presented in Table 4 and Table 5 are as follows; corrosion potential (Ecorr), which represents the potential at which the anodic and cathodic reaction rates are equal; corrosion current density (Jcorr—the anodic current density at Ecorr) as a measure of the rate of corrosion (vcorr); polarization resistance (Rp); Tafel slopes of cathodic and anodic branch were used and both Tafel slopes (βc and βa); passive domain Epas (for which the anode current density is maintained almost constant); and breakdown potential (Ebd) obtained when a transpassive region was initiated and the anodic current density increased rapidly.

**Figure 12:**
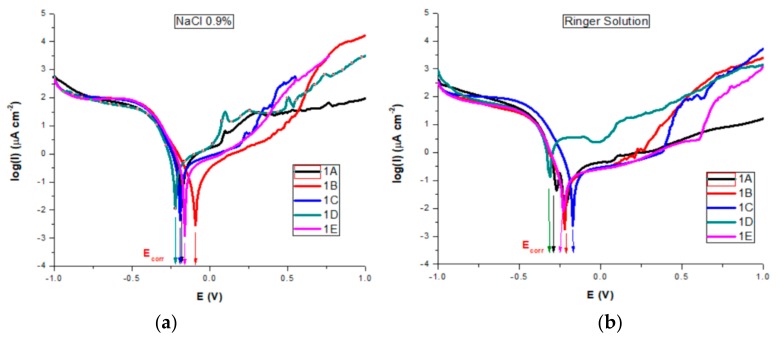
Evans diagrams in: (a) NaCl 0.9%; (b) Ringer’s solutions.

All corrosion parameters for all studied AISI 316 stainless steels in both saline solutions were determined from the corresponding polarization curves and Evans diagrams (Figure 12a,b).

The corrosion rate, vcorr, in mm/year was calculated using the PSTrace 5.5 software, where the equivalent weight (EW) 55.85 g/mol, the density (d) of 8 grams/cm^3^, and the sample area (A) in0.785 cm^2^ of the sample combined with a constant K = 3272 mm/(A cm year) (ASTM Standard G102-89, Vol.3.02, 2006).

The potentiodynamic tests on both untreated and heat-treated AISI 316 modified stainless steels in both saline solutions showed similar polarization behaviors, which is typical for the localized corrosion of stainless steel [23]. These tests performed in the two saline solutions simulating biological fluids lead to the conclusion that the Ebd and Epass are related to critical conditions which depend on the chemistry inside the pits and on their geometry. Yi and its collaborators [23] introduced a new parameter, the critical electric charge density, Qc, representing the pitting resistance of a material under test conditions:

(1) $$Q_{c}=J_{\mathrm{corr}}\times \Delta t_{p}=J_{\mathrm{corr}}\times \frac{E_{\mathrm{pass}}}{v},$$

We used the following formula to calculate Qc, considering the Jcorr and Epass values obtained from potentiodynamic tests and a 0.002 V/s scan rate (Table 6). 

The metal ions released from the samples combine with chlorides in the solution, forming metal chloride salt; thus, Qc may be interpreted as a critical cumulative charge density inside occluded pits, which causes the critical chemistry for the salt film formation. Thus, it can be observed that the smallest values of Qc were obtained for the two samples B and E, proving that these samples are more resistant to the corrosion process.

The experimental results obtained from EIS measurements are summarized in Table 7 and Table 8. This impedance technique has the advantage of using only very small signals which do not disturb the electrode properties to be measured, and which allows the determination in one experiment of double layer capacitance and polarization resistance [23].

The electrochemical impedance values were recorded at 0 V vs.Ag/AgCl, an rms perturbation of 10 mV was applied over the frequency range (f) 50 kHz to 0.1 Hz, with 10 frequency values per frequency decade and the corresponding impedance spectra are presented as Nyquist and Bode plots. The inset presents the electrical equivalent circuit used to obtain the electrical parameters by fitting experimental EIS spectra that can be used to describe the electrical features of the electrochemical interfaces between the samples and the electrolytes. This consists of the solution (cell) resistance, R1, in series with one or two parallel R–CPE configurations, which are attributed to the electrode (AISI 316 stainless steel samples), the passivation layer formed on its surface and the charge transfer process. R represents the charge transfer resistance of ions through electrochemical interfaces. The capacitance is presented through a constant phase element (CPE, ZCPE = 1/Q(iω)^n^). It was modelled as a non-ideal capacitor of capacitance Q (characterizing the double layer capacitance at the electrochemical interfaces and charge accumulation) and roughness factor n, (n = 1 for a perfectly smooth surface), ω = 2πf. For the configuration with two parallel R–CPEs of the electrical equivalent circuit, the group (R2CPE1) has been used, corresponding to an intermediate and low frequency, LF, where as the parallel group (R3CPE2) was assigned to the high frequency region, HF.

**Figure 13:**
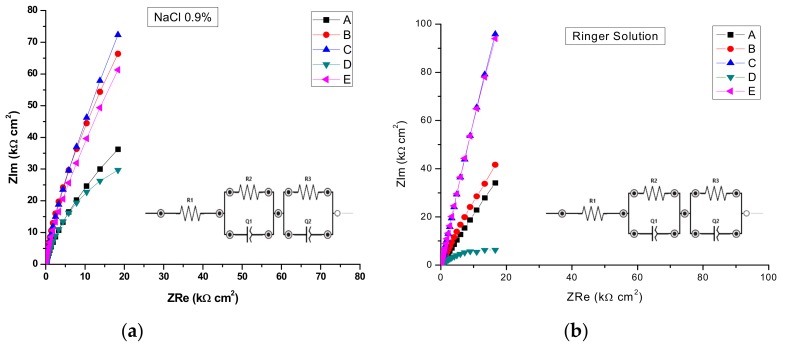
Nyquist Impedance spectra in complex plane in: (**a**) NaCl 0.9%; (**b**) and Ringer’s solution.

**Figure 14:**
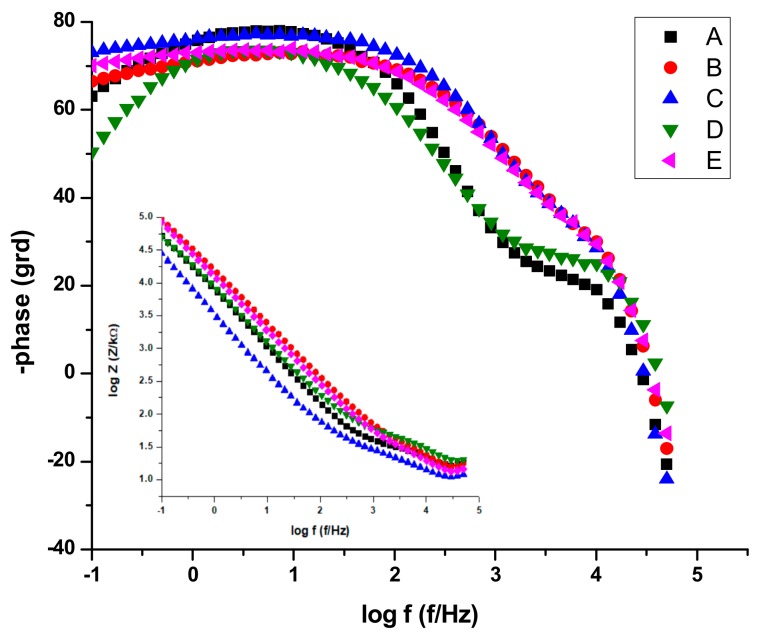
Bode plots for all stainless-steel samples in NaCl 0.9%.

By analyzing the EIS data, the Nyquist plots (Figure 13) and the Bode plots (Figure 14), the corrosion mechanism of the system can be identified.

In the above paragraph describing the potentiodynamic studies, the existence of a passivation layer was highlighted, which formed on the surface of some samples when electrochemical measurements were performed. Thus, we can conclude that R is describing the charge transfer through the (passivated) electrode–electrolyte interface, where as Q and n characterize the double layer capacitance and roughness/porosity of this interface.

The data presentations in Bode plots (Figure 14) for NaCl 0.9% (similar plots obtained for Ringer’s solution not presented) give direct information about impedance, frequency, and phase, which helps ascertain the different constituent phases of the system more easily and the information was correlated with data from the Nyquist plots. In all experimental situations, the Bode plots show that, in the high frequency region, a capacitive behavior is predominant with slightly increased values of capacitance (Q) for NaCl as compared with Ringer’s solution measurements (Table 5 and Table 6). 

The same capacitive behavior was noted for the low frequency region, with a resistive behavior in the intermediate frequency range. The slopes of the log Z against log f curves are −0.86, possibly due to the electrode surface roughness of interfacial phenomena. From the EIS measurements, we can conclude that samples C, E, and B are more resistant to the corrosion process in both NaCl 0.9% and Ringer's solutions.

## 4. Discussion

The microstructural analysis performed before and after the heat treatments with solar energy revealed changes in terms of the shape of intermetallic precipitates, which separated from the metallic matrix. As the tempering temperature increases, the carbides become spherical and larger (thickness of ~5 μm at 150 °C, 10 μm at 250 °C, and 20 μm at 350 °C, respectively). The carbides mainly precipitate at the interface of the delta ferrite phase. The rounding and coalescence of the carbides that are separated from the metal matrix has a significant effect on the mechanical strength, as evidenced by the microhardness measurements. A hardness value almost equal to the as-cast sample is noted for the sample, which was immersion quenched followed by tempering at 350 °C (E sample).This behavior could be explained by the massive separation from the solid solution of the carbides, as well as by the phenomenon of growth, coalescence and rounding of these carbides.

Thermodynamic information regarding the sample surfaces reactivity during the corrosion process is obtained by comparing the measured corrosion potential with the standard redox potential for iron (−0.440 V). According to the literature, in chloride-containing solutions, oxy-chloride compounds are resulting as pitting corrosion mechanisms [21,22,23]. The chloride ions act as activators of anodic reaction resulting in a shift to more negative values of the Ecorr of metallic samples. If the corrosion potential is dropping, it means that the sample is oxidizing and negative charges are accumulating in it. We can observe that for all samples the corrosion potential Ecorr is increased (anodic shifted) by comparison with the standard redox potential of iron, proving that their corrosion does not involve the oxidation of iron. However, an adherent, free of discontinuities oxide film (passivation film) may form in some situations on the surface.

For both salt solutions measurements, the differences between no treatments and different types of heat treatments led to a variation in all corrosion parameters. In the NaCl solution, we could see (Table 4) that sample B is characterized by the highest value of corrosion potential of −0.082 V, where as the smallest was −0.221 V for sample D. The corrosion current density drops for all samples, the biggest dropping by 3 times being noted for sample B (0.32 μA/cm²) and sample E (0.46 μA/cm²) as compared with the untreated sample A (1.01 μA/cm²). Similarly, decreases of the corrosion current densities mean that the corrosion rate is significantly reduced. Thus, for the corrosion rate, the general tendency of diminution was observed, with the smallest value of 0.007 mm/year for sample B and of 0.010 mm/year for sample E. A slight decrease in the cathodic Tafel slope, βc, was observed for treated samples as compared with the untreated sample, suggesting that the type of treatment does not modify the cathodic process mechanism. The influence of heat treatments on the anodic Tafel slope, βa indicates a modification in the mechanism of the corrosion process (anodic reaction). A general decrease was observed as compared with the value of βa for the untreated sample (0.898 V/decade) and the range of variation is related to the sample surface homogeneity and the composition obtained by different types of heat treatments. The highest drop was obtained for sample 1B (0.504 V/decade). When the anodic current increased smoothly, there was no tendency to form the passivation layer. An overall decreasing of Epass and Ebd for almost all heat-treated samples was observed, noting a drop of Epass from 1.25 V for the untreated sample to 0.55 V and a shift for Ebd from 1.00 V to 0.30 V for sample C. The pitting corrosion creates some current spikes in the polarization curve, as can be observed for samples A and D, which are the proof of the formation of pits with a short lifetime. Although the untreated sample A presents the larger passive domain and the highest breakdown potential, the corrosion current density and potential, as well as the corrosion rate have also the highest values. The high values of Ebd and Epass for A can be explained by the formation of pits with short lifetime which passivate again. A comparison of all values reveals that samples B and E exhibit a significant improved pitting corrosion resistance in NaCl 0.9% versus the other heat-treated samples.

In Ringer’s solution containing more ions besides Na, stainless steel samples rapidly corrode and form an oxide (passivation) layer, all Ecorr values for both untreated and heat-treated samples being cathodically shifted compared to those obtained in NaCl, except sample C (−0.172 V). Jcorr had smaller values for all samples than those obtained in NaCl. As compared with the untreated sample (0.39 μA/cm²), for the heat-treated samples the Jcorr values were also smaller, with a higher decrease for sample 1B, similar value for samples C and E and with a high increase for D. Similar tendencies were noted for the other corrosion parameters. Pitting corrosion creates some current spikes in the polarization curve, as can be observed, to a lesser extent, for all samples. However, that happens for samples A and D. For those samples, the plots show a short decrease in the current density, followed by an almost linear increase when the potential is increased towards anodic potentials, suggesting free corrosion behavior, with no evidence of a passivation layer forming on the sample surface. However, we can say that, similarly to the NaCl 0.9% measurements, these spikes are the result of the formation of pits which passivate again, so the pits have only a short lifetime. The existence of a passivation layer forming on sample surfaces is suggested by the both Epass and Ebd values, which depend on the heat treatment type. The untreated sample A presents the larger passive domain and the highest breakdown potential, but also the highest values for both corrosion current density and potential, suggesting also the formation of pits with a short lifetime which passivate again. The treated sample E presents the highest values for Epass and Ebd, followed by sample B. Comparing all obtained values we can also conclude that samples B and E are more resistant to the corrosion process in Ringer’s solution, followed by samples C and D.

A circuit with one parallel R-CPE element in series was necessary to fit the data in the case of the untreated sample A, and sample D heat-treated in NaCl, where as in Ringer’s solution it was used just for sample D. Thus, sample D, in both solutions was characterized by different corrosion behavior, with no evidence of passivation layer forming on the sample surface. However, taking into account the findings in potentiodynamic measurements for sample D, we can also conclude that pits are formed with short lifetime which passivate again. For both saline solutions, the charge transfer resistance R2 of sample D had the smallest value, as compared with all samples, indicating a charge transfer and suggesting the formation of pores directly on the surface of the sample. The situation is more obvious in the case of the Ringer’s solution, when values of 21.88 kΩ cm^2^ for R2 and 0.703 for n1 have been noted as compared with 791.28 kΩ cm^2^ and 0.843 for sample C. Thus, the high values of corrosion current and corrosion rate from Table 5 can be correlated with the smallest value of index n, indicating a very inhomogeneous surface due to the in-depth growth of pores, which would allow the electrolyte to penetrate the sample surface and spread over it. In the rest of the situations, a circuit with two parallel R-CPE elements in series was used, indicating a charge transfer process controlling the corrosion of the stainless steel due to the formation of pitting pores in the passivation layer on top of the samples and to the charge accumulation at the interfaces. 

A similar discussion as for sample D can be conducted for sample A, noting that in Ringer’s solution, a very nonuniform corrosion layer is formed on the surface: low R2 and R1 values correlated with small values of n2 and n1 indicate the in-depth growth of pores that will allow the electrolyte to penetrate the layer and eventually reach the electrode surface and spread over it. From the other heat-treated samples in EIS experiments, sample C shows a better protection at corrosion since both the charge transfer resistance R2 and the roughness indexes, n, have the highest values. That indicates a low degree of pores formation in the passivated layers, for both saline solutions, which will not allow the electrolyte penetration to the sample surface. Similar behavior can be noted for sample E. 

## 5. Conclusions

The obtained results accredit the idea that a better absorption of the thermal energy from the concentrated solar energy can be obtained, in the case of heat treated samples with reduced surfaces. The phase transformations which occurred in a very short time in the analyzed alloy, led to the improvement of structural and corrosion resistance characteristics. After heat treatments, the modified AISI 316 stainless steel samples show a microstructure consisting of austenitic matrix (Feγ) with chromium-rich and molybdenum-rich fine complex carbides (K), M_7_C_3_ (CrFe)_7_C_3_-typeand M_23_C_6_ (CrFeMo)_23_C_6_-type, and also small quantities of delta ferrite (Feδ). Furthermore, there has been noted that chains of large carbides are displayed at the boundaries of the austenite grains, while a few smaller carbides are located at the interior of the grains. By increasing the tempering temperature from 150 °C to 350 °C, the morphology of the carbides changes from small to coarse, with dimensions ranging from 5 to 20 microns. 

The sample heat-treated by immersion quenching followed by tempering at 350 °C (E) stands out due to superior values of average microhardness (261,73 HV0.1/10). The superior values of the hardness are attributed to the phenomenon of growth, coalescence and rounding of the carbides precipitated in the matrix as a result of the heat treatment.

Potentiodynamic measurements conclude that AISI 316 modified stainless steel samples E and B are more resistant to the corrosion process in both NaCl 0.9% and Ringer’s solution, which simulate biological fluids. EIS measurements concluded also that samples E and B exhibit a good behavior during corrosion in saline solutions, along with sample C. For these samples, the existence of a passivation layer which forms on their surface has been highlighted by both electrochemical studies. Sample D and, to a lesser extent, sample A, in both solutions, are characterized by a behavior similar to the free corrosion behavior, and all measurements suggest the formation of pits with short lifetime which passivate again on the sample surface. 

## Figures and Tables

**Table 1:** Chemical composition of 316 AISI modified stainless steel (wt%).

C	Si	Mn	P	S	Cr	Ni	Mo	Cu	Fe
0.07	1.40	1.06	0.004	0.035	20.50	12.40	2	0.26	Bal.

**Table 2:** Heat treatment parameters for modified 316 AISI samples.

Sample	Heat Treatments					
	Quenching			Tempering		
	T_A_ (°C)	τ_A_ (min)	Cooling	T_T_ (°C)	τ_T_ (min)	Cooling
A untreated	-	-	-	-	-	-
B	1050	10	H_2_O	-	-	-
C	1050	10	H_2_O	150	10	Air
D	1050	10	H_2_O	250	10	Air
E	1050	10	H_2_O	350	10	Air

Remark: T_A_: austenitization temperature; τ_A_: austenitization holding time; T_T_: tempering temperature; τ_T_: tempering holding.

**Table 3:** Average HV_0.1/10_ hardness for the five samples.

Sample	HV _0.1/10_Mean (0.980665 N)	Standard Deviation
A	303.433	14.029
B	225.316	34.288
C	228.706	22.037
D	226.368	32.856
E	261.736	34.586

**Table 4:** Corrosion parameters in NaCl 0.9%.

Sample	Ecorr (V)	Icorr (μ)A	Jcorr (μA/cm²)	Rp (kΩ)	βa (V/decade)	βc (V/decade)	vcorr (mm/year)	Epass (V)	Ebd (V)
1A	−0.181	0.791	1.01	85.00	0.898	0.187	0.023	1.25	1.00
1B	−0.092	0.253	0.32	21.60	0.504	0.168	0.007	0.75	0.50
1C	−0.191	0.437	0.56	101.60	0.861	0.116	0.013	0.55	0.30
1D	−0.221	0.514	0.65	81.60	0.593	0.115	0.015	0.90	0.55
1E	−0.161	0.359	0.46	130.00	0.643	0.129	0.010	0.63	0.35

**Table 5:** Corrosion parameters in Ringer’s solution.

Sample	Ecorr (V)	Icorr (μA0	Jcorr (μA/cm²)	Rp (kΩ)	βa (V/decade)	Βc (V/decade)	vcorr (mm/year)	Epass (V)	Ebd (V)
1A	−0.221	0.306	0.39	178.90	1.175	0.141	0.009	1.35	1.05
1B	−0.221	0.231	0.29	207.40	1.038	0.123	0.007	0.72	0.35
1C	−0.171	0.267	0.34	167.00	1.041	0.114	0.008	0.68	0.43
1D	−0.311	2.849	3.63	25.00	10.44	0.167	0.083	0.80	0.45
1E	−0.231	0.275	0.35	172.00	1.693	0.117	0.008	1.00	0.60

**Table 6:** Critical electric charge densities, Qc, in NaCl 0.9% and Ringer’s solution.

Qc, (μC/cm²)					
Sample	A	B	C	D	E
NaCl 0.9%	631.25	120.00	154.00	292.50	144.90
Ringer’s solution	263.25	104.40	115.60	1452.00	175.00

**Table 7:** Electrochemical impedance spectroscopy (EIS) parameters in NaCl 0.9%.

Sample	R 1	R 2	Q 1	n1	R 3	Q 2	n2
	(Ω cm^2^)	(kΩ cm^2^)	(μF cm^−2^ s^n−1^)	-	(kΩ cm^2^)	(μF cm^−2^s^n−1^)	-
A	15.89	170.35	33.26	0.847	-	-	-
B	12.61	2780.84	19.54	0.802	1.27	113.50	0.702
C	9.80	3055.22	22.50	0.840	17.62	137.45	0.997
D	19.52	91.06	29.94	0.826	-	-	-
E	11.36	1566.86	27.38	0.814	76.54	117.30	0.819

**Table 8:** EIS parameters in Ringer’s solution.

Sample	R 1	R 2	Q 1	n1	R 3	Q 2	n2
	(Ω cm^2^)	(kΩ·cm^2^)	(μF cm^−2^ s^n−1^)	-	(kΩ cm^2^)	(μF cm^−2^s^n−1^)	-
A	10.34	226.43	36.60	0.735	21.03	10.73	0.798
B	8.49	307.25	45.50	0.882	28.60	37.03	0.784
C	8.05	791.28	14.03	0.843	9.26	53.17	0.818
D	9.73	21.88	76.82	0.703	-	-	-
E	7.19	636.47	13.54	0.849	6.59	40.62	0.851
